# Reference genes identification for normalization of qPCR under multiple stresses in *Hordeum brevisubulatum*

**DOI:** 10.1186/s13007-018-0379-3

**Published:** 2018-12-18

**Authors:** Lili Zhang, Qike Zhang, Ying Jiang, Yang Li, Haiwen Zhang, Ruifen Li

**Affiliations:** 1 Beijing Key Laboratory of Agricultural Genetic Resources and Biotechnology, Beijing Agro-biotechnology Research Center, Beijing Academy of Agriculture and Forestry Sciences, Beijing, China; 2 College of Life Science, Hebei Normal University, Shijiazhuang, China

**Keywords:** *Hordeum brevisubulatum*, Reference gene, Abiotic stress, Quantitative real-time PCR

## Abstract

**Background:**

Real-time quantitative PCR has been widely used as the most reliable method to measure gene expression, due to its high accuracy and specificity. Wild barley (*Hordeum brevisubulatum* (Trin.) Link) is a wild relative species in Triticeae that has strong tolerance to abiotic stresses and extremely wide adaptation. However, suitable references gene have not been documented for standardization of gene expression in wild barley under abiotic stress.

**Results:**

Here we report the first systematic and comprehensive analysis of reference genes for quantitative real-time PCR standardization in wild barley. We selected 11 genes, including *ACT* (*Actin*), *ADP* (*ADP*-*ribosylation factor 1*), *CYP2* (*Cyclophilin 2*), *EF*-*1α* (*Elongation factor 1*-*alpha*), *GAPDH* (*Glyceraldehyde 3*-*phosphate dehydrogenase*), *HSP90* (*Heat shock protein 90*), *TUBα* (*Alpha*-*tubulin*), *TUBβ6* (*Beta*-*tubulin 6*), *UBI* (*Ubiquitin*), *18SrRNA*-*1* (*guanine1575*-*N7*-*methyltransferase*) and *18SrRNA*-*3* (*adenine1779*-*N6*-*dimethyltransferase*) from a wild barley transcriptome database and analyzed their expression stabilities in shoots and roots of wild barley seedling under various stress conditions using comparative ΔCt, BestKeeper, Normfinder and geNorm software. The results demonstrated that *ADP* was the most suitable reference gene in salt stress while *UBI* showed peak stability under mannitol and ABA stress; *EF*-*1α* was the most appropriate reference gene for PEG, GA_3_, ethylene and heat stress; *18SrRNA*-*3* was the best choice for cold stress; and *TUBα* was the first stable gene across different tissues.

**Conclusions:**

Our main contribution was to identify reference genes with suitable and stable expression in wild barley under various stress conditions and in different tissues to provide a useful resource for future studies. The results demonstrate the importance of transcriptome data as a useful resource for the screening of candidate reference genes and highlight the need for specific reference genes for specific conditions. Furthermore, these findings will provide valuable information for wild barley and relative species for future research.

**Electronic supplementary material:**

The online version of this article (10.1186/s13007-018-0379-3) contains supplementary material, which is available to authorized users.

## Background

Wild barley (*Hordeum brevisubulatum*), which is a relative of cultivated barley (*H. vulgare*), is an important wild germplasm resource with ecological, feeding and ornamental value. The salient feature of *H. brevisubulatum* is its high tolerance to multiple abiotic stresses, including drought, salinity and alkalinity, allowing it to grow in saline-alkali grasslands in the North of China where it is used as a major forage for livestock [1, 2]. Wild barley has evolved molecular mechanisms of abiotic stress tolerance during long-time adaptation. To better understand its adaptable mechanisms, it is important to discover key abiotic stress genes and dissect their function; gene expression profiles are, therefore, the first step for gene functional analysis. Although there were several reports on ion balance and gene function of *H. brevisubulatum* [3], appropriate reference genes for multiple stresses have not been documented yet.

Quantitative real-time PCR (qRT-PCR) is a commonly used technique for investigating gene expression levels with high accuracy and sensitivity [4, 5]. Approaches for detection of the amounts of PCR products (amplicons) using qRT-PCR are classified into two categories: relative quantification based on housekeeping genes (HKGs) and absolute quantification achieved with DNA standards via calibration curves [6]. One of the most straightforward and robust methods for accurately quantifying subtle changes is the relative quantification. However, gene expression can be affected by many confounding factors such as RNA extraction, reverse transcription and qRT-PCR efficiency [7, 8]. To avoid biased results and erroneous interpretations, a critical component of relative quantification analysis is the normalization of data by measuring in parallel the expression of HKGs that are commonly used as “reference genes” from the same specimen [9–12]. The expression of HKGs that are constitutively expressed to maintain cellular function is relatively steady in different tissues and organs of specimens under various biotic and abiotic circumstances [13]. Next-generation sequencing (NGS) data mining and HKG identification in model species have shown that some internal controls exhibited both species- and tissue- specific expression patterns. Furthermore, their expression levels are also influenced by environmental factors (drought, salinity, temperature and hormones) as well as specific experimental conditions [6, 14–17].

In general, genes that play key roles in the maintenance of basic cellular functioning are typically selected as reference genes such as *18S ribosomal RNA* (*18SrRNA*), *beta*-*actin* (*β*-*actin*), *elongation factor*-*1 alpha* (*EF*-*1α*), *ubiquitin* (*UBI*) and *glyceraldehyde*-*3*-*phosphate dehydrogenase* (*GAPDH*), which have been widely adopted for normalization [18, 19]. Nevertheless, rather than randomly selecting reference genes from various sources, the reference genes used in specific species require experimental validation. Moreover, single gene quantification qPCR assays are well known to frequently exhibit variability in gene expression under various experimental conditions [12]. The Minimum Information for Publication of Quantitative Real-Time PCR Experiments (MIQE) guidelines developed for the proper selection and validation of stable candidate reference genes for qPCR experiments highly recommend average data from more than two reference genes [20–22]. Several statistical algorithms such as geNorm, NormFinder and BestKeeper, have been developed for the selection of reference genes for qRT-PCR analysis [17, 23, 24]. These tools can calculate the expression stability value (M) for different reference genes. Application of all these algorithms can contribute to the identification of the best stable reference genes for different experimental samples.

In this study, we investigated several reference genes based on *H. vulgare* and *Pennisetum glaucum* [25–29]. After extracting the corresponding HKGs from *H. brevisubulatum* transcriptome data and detecting their expression using qRT-PCR, we identified 11 candidate reference genes and measured the expression stability of these genes in different adult tissues or under various abiotic stress and hormone treatments then analyzed the qRT-PCR results using GeNorm [23], NormFinder [17], BestKeeper [24] and the comparative ΔC_t_ method [30]. The web-based comprehensive tool RefFinder was used to rank their expression stability [31]. This research represents the first comprehensive systematic screening of reference genes for *H. brevisubulatum* based on experiments examining temporal and spatial expression in response to abiotic stress and hormone treatment. Furthermore, the results will improve the accuracy and reliability of the qRT-PCR technique and will provide a useful reference for gene expression studies in specific species in the *Hordeum* genus.

## Results

### Validation of candidate reference genes from transcriptome data

Based on previous research in *H. vulgare* [28, 32], *Pennisetum glaucum* [25] and *Corchorus capsularis* [27], we screened 15 candidate reference genes in the transcriptome libraries of *H. brevisubulatum* and found four candidate genes that were weakly expressed or showed no specific amplification in different tissues but passed the BLAST test. Further screening identified 11 other candidate reference genes with effective specificity and amplification: *ACT* (*Actin*), ADP (*ADP*-*ribosylation factor 1*), CYP2 (*Cyclophilin 2*), EF-1α (*Elongation factor 1*-*alpha*), GAPDH (*Glyceraldehyde 3*-*phosphate dehydrogenase*), HSP90 (*Heat shock protein 90*), TUBα (*Alpha*-*tubulin*), TUBβ6 (*Beta*-*tubulin 6*), UBI (*Ubiquitin*), 18SrRNA-1 (*guanine1575*-*N7*-*methyltransferase*) and 18SrRNA-3 (*adenine1779*-*N6*-*dimethyltransferase*). The specific amplification primers of these 11 candidate reference genes for qRT-PCR are shown in Table 1. PCR amplification efficiencies ranged from 0.88 to 1.15; coefficients of determination (R^2^) based on linear regression varied from 0.991 to 1.000.

**Table 1:** Comprehensive details of 11 candidate reference genes used for normalization

Gene symbol	Primers (5′–3′) Forward/reverse	Length (bp)	T_m_ (°C)	PCR efficiency	Regression coefficient (R^2^)
*ACT*	TGCATGGTGTTCTCTGCACT GCCAAAGCGTGATACTGTCG	121	60	0.92	0.997
*ADP*	CAAGAGATAGTATGTGTGCGTATG AACCACGGCACAGAAATACTGAT	115	60	1.03	0.997
*CYP2*	CCTGTCGTGTCGTCGGTCTAAA ACGCAGATCCAGCAGCCTAAAG	151	60	0.96	0.991
*EF*-*1α*	CAACAACGCCCAGGAACAAC AGAAGAAGGACCCCACTGGT	159	60	0.94	0.994
*GAPDH*	AGCTGCACCACTAACTGCCT AACAGTGGTCATCAAACCCTCAAT	84	60	0.96	0.997
*HSP90*	CAGCACCTCCTTGATGACCTT GGAGTTTGAGGGCAAGAAGC	136	60	0.97	0.997
*TUBα*	CGAGATGCACTCCCTCATGG CGTCTTCGTACTCGCCTCTC	128	60	1.15	1.000
*TUBβ6*	CCCGACGAGAACGCTTCAAT CTGGATGTGCAGGATCTCCC	74	60	0.89	0.996
*UBI*	TGGATGTTGTAGTCGGCGAG ACGTCAAGGCCAAGATCCAG	112	60	0.90	0.996
*18SrRNA*-*1*	CCCGCTTCTAAGTCGGTGTT CAACAGAAGCGCGATACAGC	99	60	0.88	0.997
*18SrRNA*-*3*	TTTCGTGAGGGCCTGCTTAG GACTCACAGAACATGGGGCA	83	60	0.97	0.997

The correlation coefficients (R^2^) and slope values were obtained from the standard regression curves and the PCR amplification efficiencies (E) were calculated according to the following equation: E = (10^−1/slope^ − 1)

### Quality control

**Fig. 1:**
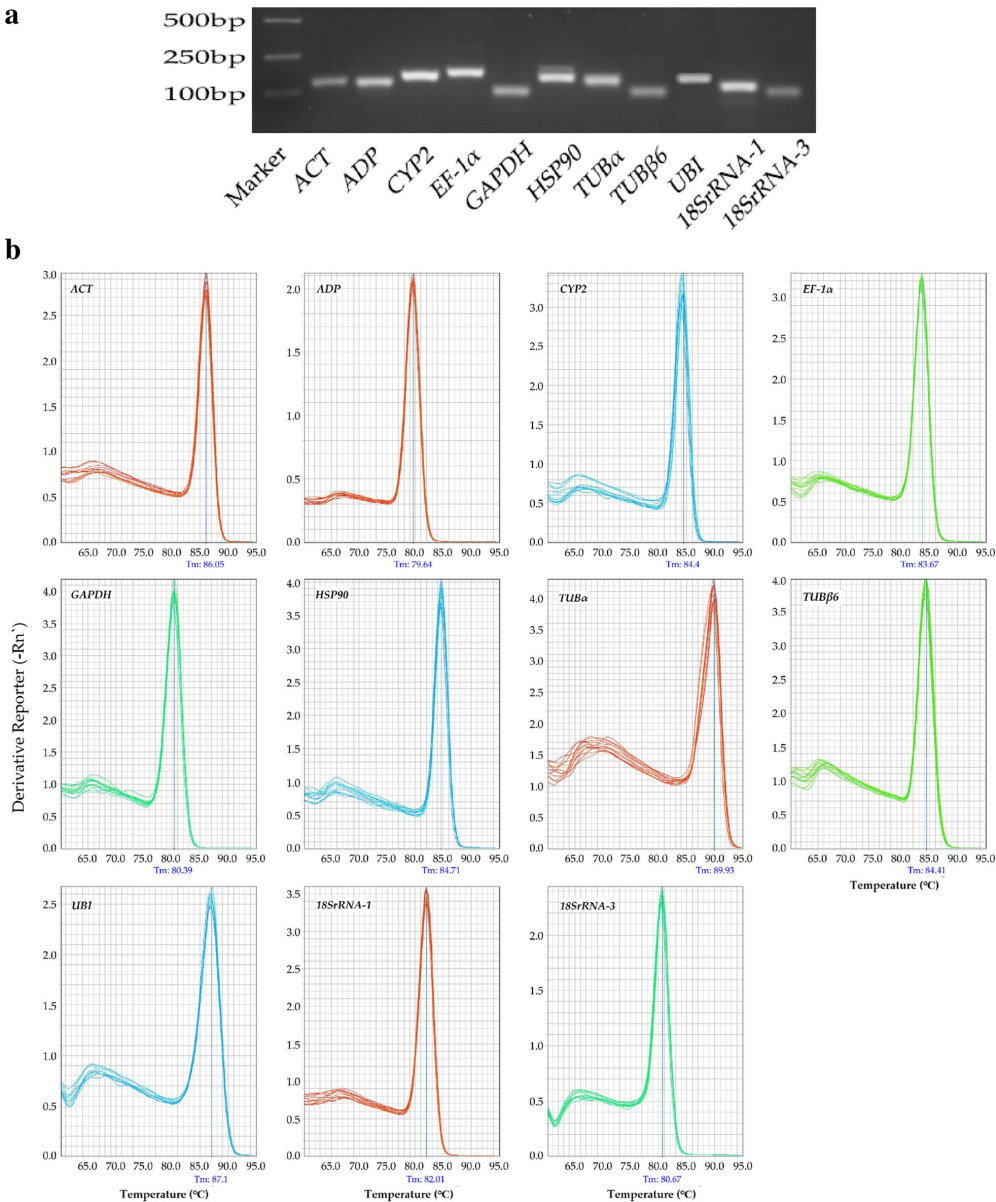
Specificity of each candidate reference gene primer pair. **a** Confirmation of the specificity of qPCR primer amplification of candidate reference genes by agarose gel electrophoresis. **b** Melting curve analysis of quantitative real-time PCR (qRT-PCR) amplification of 11 candidate reference genes in *H. brevisubulatum*

We sampled the shoot and root parts of *H. brevisubulatum* seedlings separately and extracted total RNA using Trizol (Takara, Dalian, China). A NanoDrop2000 spectrophotometer (Thermo Fisher Scientific Inc., Waltham, MA, USA) was used to measure RNA concentration; acceptable RNA quality was defined as an OD_260_/OD_280_ ratio of between 1.8 and 2.0 and an OD_260_/OD_230_ ratio of > 1.7. As shown in Fig. 1a, the specificity of each single PCR product was confirmed by 1.2% agarose gel electrophoresis, with samples matched with their predicted product sizes. The melt curves of the 11 candidate reference genes showed a single peak in each case, reflecting their stability and specificity (Fig. 1b) [21]. This laid a foundation for the accuracy of the experimental results.

### Expression patterns of candidate reference genes

**Fig. 2:**
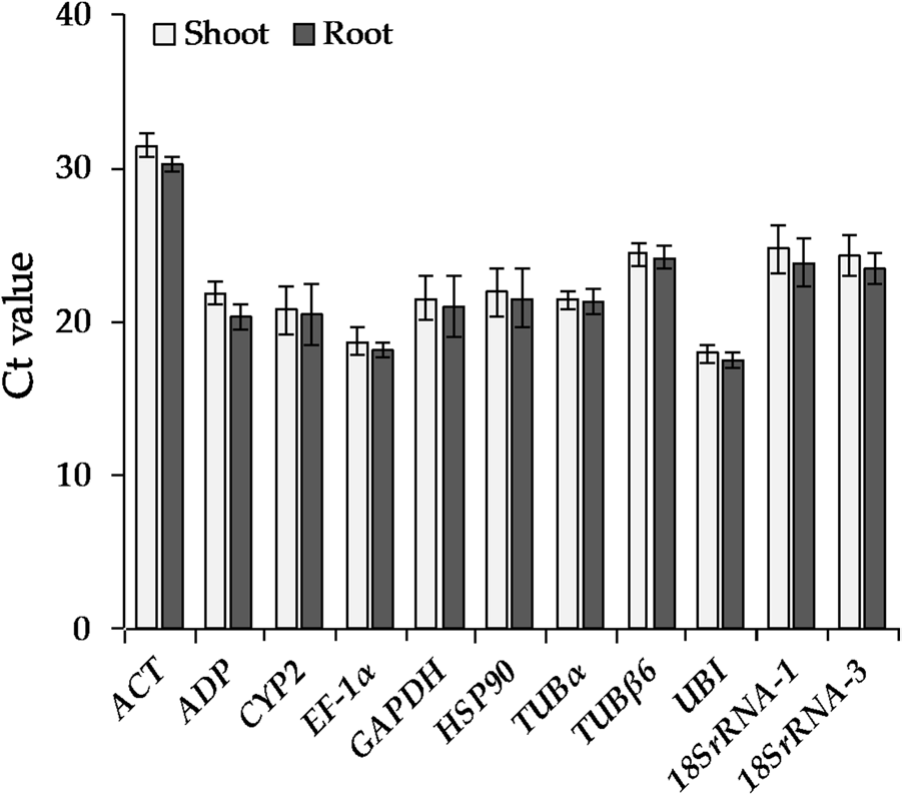
Comparative analysis of Ct values of 11 reference genes in shoot and root tissue under normal conditions. The data represent the mean ± SD

**Fig. 3:**
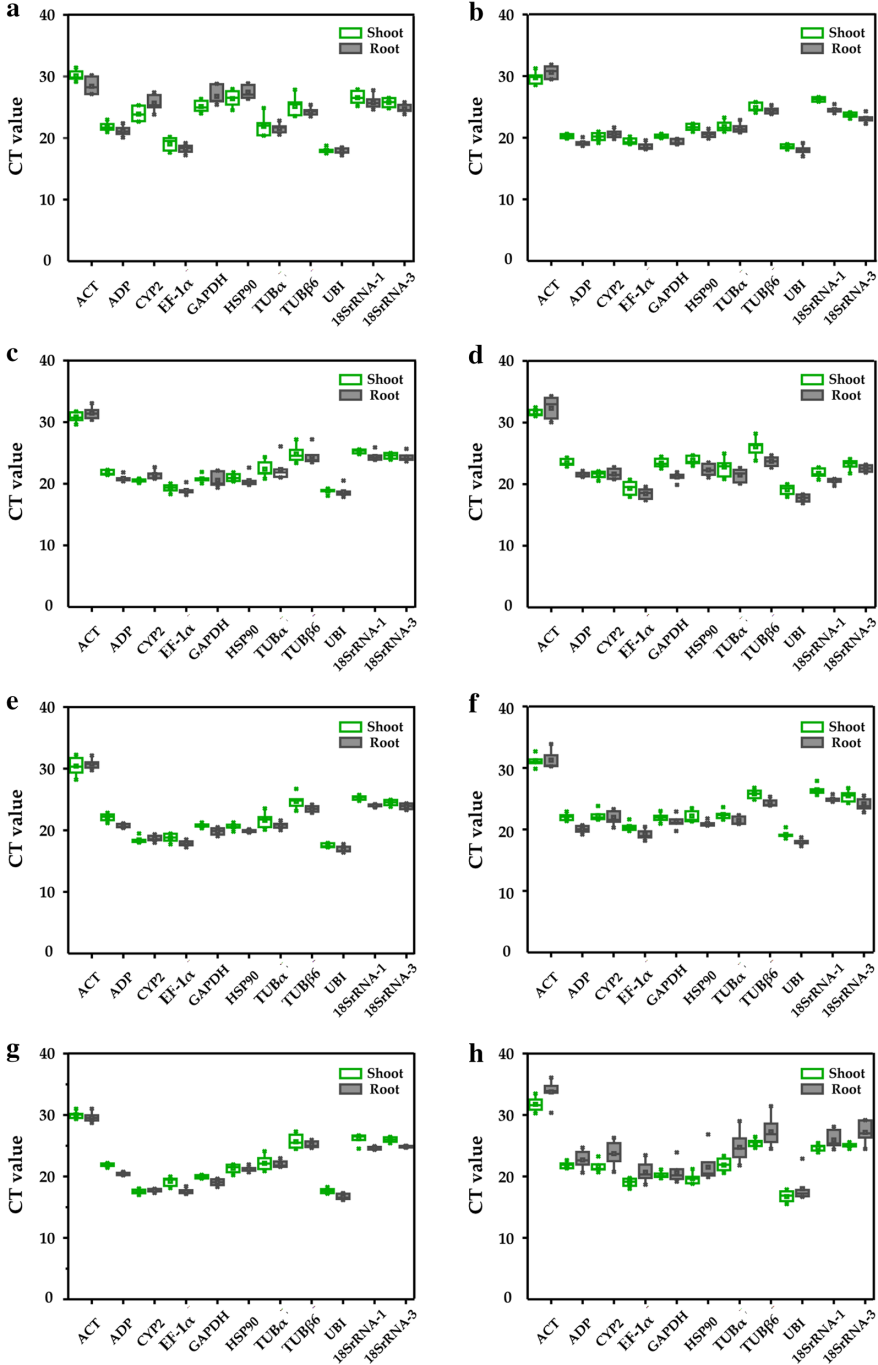
Boxplot showing the variation in CT values of candidate reference genes in different treatments and tissues. **a** 350 mM NaCl treatment, **b** 10% PEG6000 treatment, **c** 350 mM mannitol treatment, **d** 20 μM ABA treatment, **e** 100 μM GA_3_ treatment, **f** 100 μM ethylene treatment, **g** 4 °C cold stress, and **h** 42 °C heat stress. The boxes indicate the first and third quartile, while the middle line marks the median, and points represent the average. The whisker caps show the distribution of the highest and lowest CT values, and the farther points represent outliers

We acquired and analyzed the cycle threshold (Ct) values that were generated from qRT-PCR of 11 candidate reference genes and presented the variation of all samples under each treatment. The Ct values of the 11 reference genes were very similar before treatment in both shoot and root with no significant changes (Fig. 2). The expression of candidate reference genes under each abiotic stress was assessed using the change in Ct values at different time points (0 h, 0.5 h, 1 h, 2 h, 3 h, 6 h, 12 h) in shoot and root tissues (no 12 h sample was included for ethylene treatment; Fig. 3). The results indicated that these internal reference genes maintained relatively stable Ct values under various treatments, with the exception of some separate outliers in roots under heat stress (Fig. 3h). With increasing treatment times there were some fluctuations in the Ct values of several reference genes under certain treatments as shown in the line chart depicted in Additional file 1: Fig. S1.

A high Ct value represents a low expression level [21]; of the 11 reference genes, *EF*-*1α* and *UBI* had the highest expression levels and *ACT* the lowest levels (Fig. 3). In NaCl, the expression of *ACT* in roots was higher than shoots while the expression of *CYP2* and *GAPDH* in roots were lower than shoots (Fig. 3a). In PEG6000, the expression of *ACT* in roots was lower than shoots and the expression of *18SrRNA*-*1* in roots was higher than shoots (Fig. 3b). In ABA, the expression of *ADP*, *GAPDH*, *HSP90* and *TUBβ6* in roots was higher than shoots (Fig. 3d). In ethylene, the expression of *ADP* in roots was higher than shoots (Fig. 3f). In cold, the expression of *ADP* and *18SrRNA*-*1* in roots was higher than shoots (Fig. 3g). In heat, the expression of *ACT, CYP2*, *TUBα*, *TUBβ6* and *18SrRNA*-*3* in roots was lower than shoots (Fig. 3h). However, there was no significant difference in the expression of the 11 genes in shoots and roots under mannitol and GA_3_ treatment (Fig. 3c, e). While there was no difference in the expression of these 11 genes in shoot and root tissue under normal conditions (Fig. 2) some of these genes were differentially induced in root and shoot tissue by stress (Fig. 3). Notably, *EF*-*1α* and *UBI* did not differ significantly in shoots and roots under eight stress and phytohormone treatments, revealing the importance and stability of these two reference genes in the selection of internal reference genes in *H. brevisubulatum*.

### Stability analysis of candidate reference genes

**Fig. 4:**
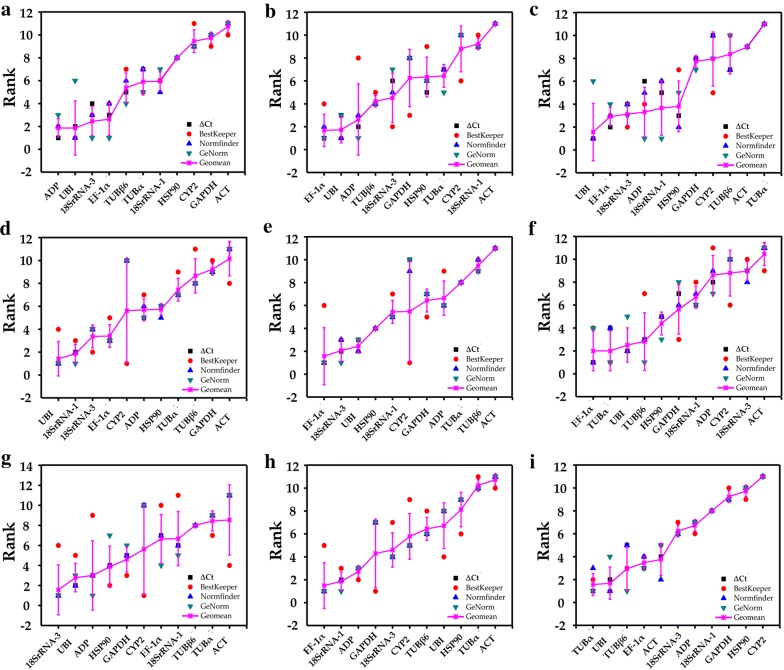
Aggregation of four algorithmic rankings of 11 candidate reference genes under various stress treatments and tissues in *H. brevisubulatum*. **a** 350 mM NaCl treatment, **b** 10% PEG6000 treatment, **c** 350 mM mannitol treatment, **d** 20 μM ABA treatment, **e** 100 μM GA_3_ treatment, **f** 100 μM ethylene treatment, **g** 4 °C cold stress, **h** 42 °C heat stress, and **i** shoot and root tissues

To explore and determine which of the 11 reference genes is suitable and stable for multiple stress treatments, we analyzed the stabilities of the 11 reference genes at different time points for each stress treatment using four commonly used methods: comparative ΔCt, BestKeeper, Normfinder and geNorm [17, 24, 30, 31]. Gene average STDEV was calculated by the ΔCt method and genes stabilities were calculated by BeastKeeper, Normfinder and geNorm (Fig. 4; Additional file 2: Table S1). We aimed to avoid any tissue-specific reference genes for each treatment so comprehensively compared the gene expression in shoot and root tissue at different time points.

#### Osmotic stress

Salt stress (NaCl treatment) and drought stress (PEG6000, mannitol treatment) both induce cell osmotic stress responses [36]. According to comparative ΔCt, BestKeeper and Normfinder, *ADP* and *UBI* were the most stable genes in NaCl treatment while *ACT*, *CYP2* and *GAPDH* were the least stable. *EF*-*1α*, *UBI* and *ADP* were the most stable genes in PEG treatment according to comparative ΔCt, Normfinder and geNorm while *18SrRNA*-*1, CYP2* and *ACT* were the most unstable. *UBI* and *EF*-*1α* were two of the three genes with the best stability in mannitol treatment according to comparative ΔCt, BestKeeper and Normfinder while *TUBβ6*, *ACT* and *TUBα* were the least stable (Fig. 4a–c; Additional file 2: Table S1).

**Fig. 5:**
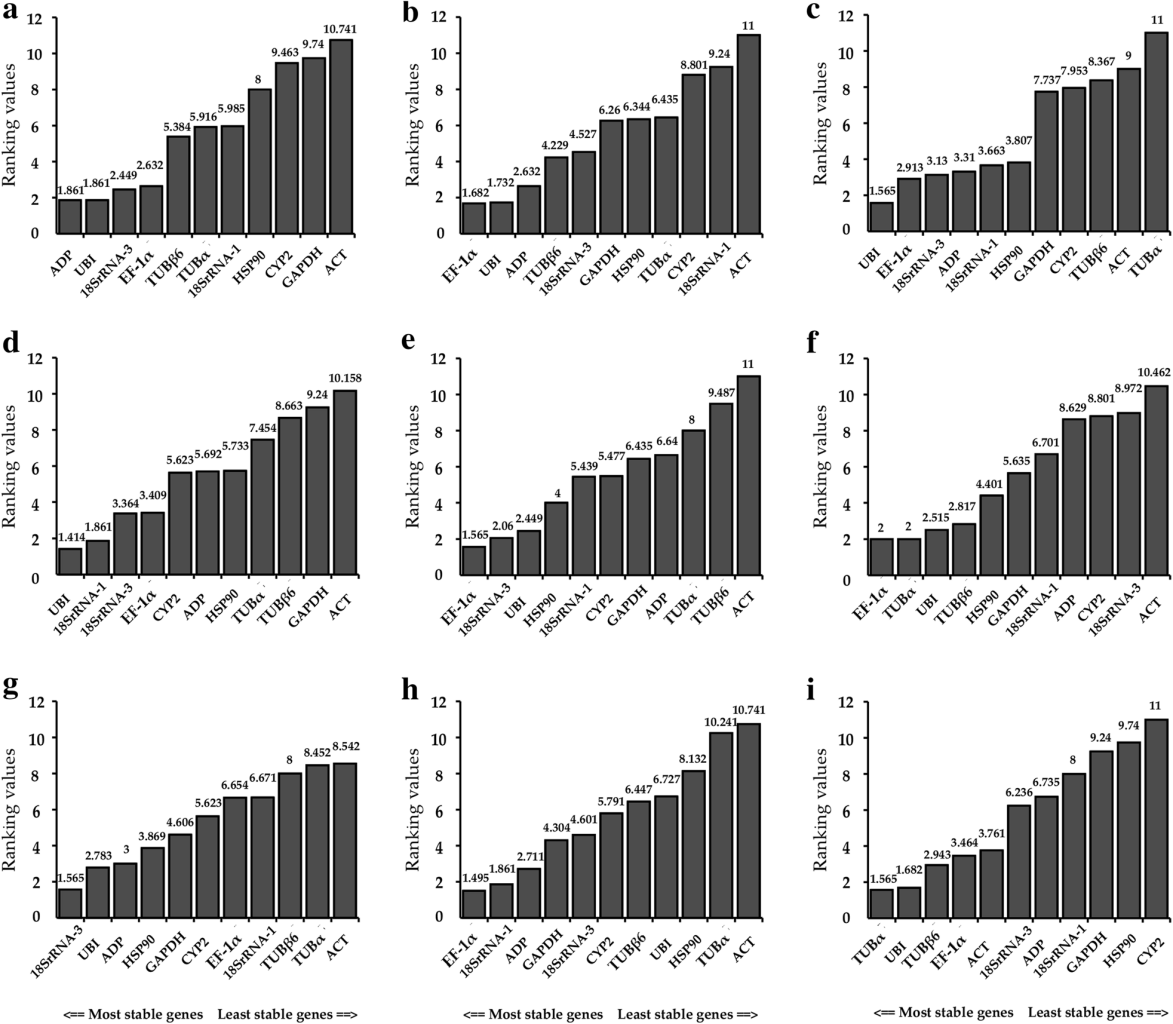
Ranking values of comprehensive gene stability of 11 candidate reference genes under various stress treatments and tissues in *H. brevisubulatum* based on the Geomean method of RefFinder and measured across **a** 350 mM NaCl treatment, **b** 10% PEG6000 treatment, **c** 350 mM mannitol treatment, **d** 20 μM ABA treatment, **e** 100 μM GA_3_ treatment, **f** 100 μM ethylene treatment, **g** 4 °C cold stress, **h** 42 °C heat stress, and **i** shoot and root tissues

**Fig. 6:**
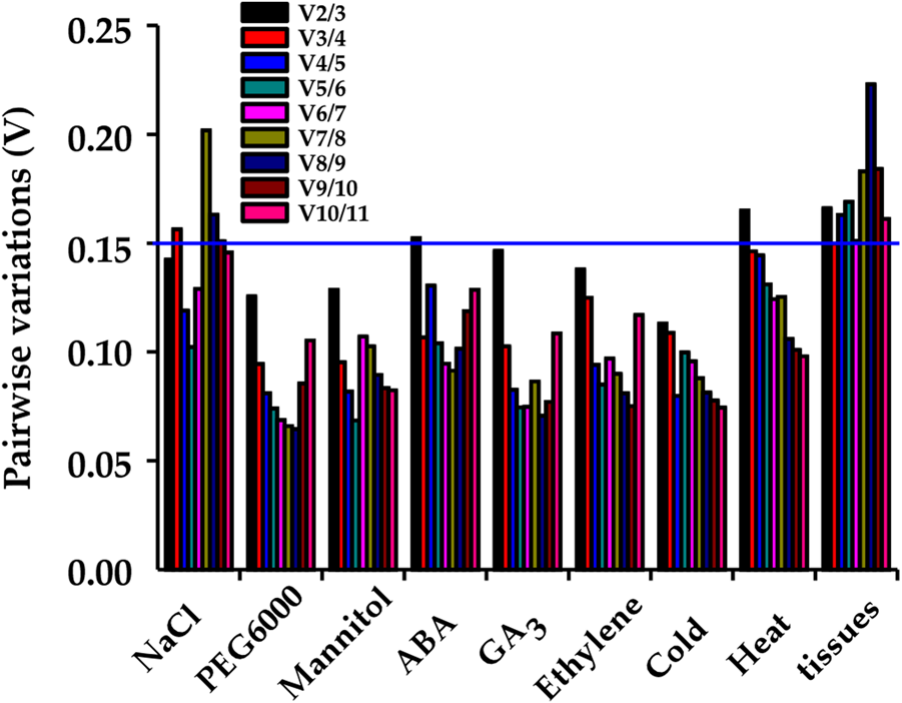
Pairwise variation (Vn/Vn + 1) analysis of the number of candidate reference genes in *H. brevisubulatum* under various stress treatments and tissues. Pairwise variation was analyzed by geNorm software that determined the optimal number of control genes for normalization. A value < 0.15 indicates that the normalization could not be dramatically changed by additional reference genes

According to RefFinder, the order of the 11 reference genes in terms of stability across NaCl treatment was: *ADP* > *UBI* > *18SrRNA*-*3* > *EF*-*1α *> *TUBβ6* > *TUBα* > *18SrRNA*-*1 *> *HSP90* > *CYP2* > *GAPDH* > *ACT*. The ranking of gene stability across PEG treatment was: *EF*-*1α* > *UBI* > *ADP *> *TUBβ6 *> *18SrRNA*-*3* > *GAPDH *> *HSP90* > *TUBα* > *CYP2* > *18SrRNA*-*1 *> *ACT*. In mannitol treatment, the order of gene stability was: *UBI* >* EF*-*1α* > *18SrRNA*-*3* > *ADP* > *18SrRNA*-*1* > *HSP90* > *GAPDH* > *CYP2 *> *TUBβ6* > *ACT* > *TUBα* (Fig. 5a–c). GeNorm analysis results showed that the pair-wise values of V2/3 were less than the cut-off value of 0.15 in any NaCl, PEG, or mannitol treatment (Fig. 6). A value of < 0.15 indicates that the supplemental reference genes will not manifestly change the normalization. Based on the RefFinder recommendations for the selection of reference genes and on the convenience of operation, *ADP* and *UBI* were considered suitable reference genes across NaCl treatment of *H. brevisubulatum* and *UBI* and *EF*-*1α* were determined suitable reference genes across both PEG and mannitol treatment (Table 2).

**Table 2:** Definitive reference genes under multiple treatments and tissues in *H. brevisubulatum*

Treatment	Optimal single reference gene	Optimal reference gene combination
NaCl	*ADP*	*ADP UBI*
PEG6000	*EF*-*1α*	*EF*-*1α UBI*
Mannitol	*UBI*	*EF*-*1α UBI*
ABA	*UBI*	*UBI 18SrRNA*-*1 18SrRNA*-*3*
GA_3_	*EF*-*1α*	*EF*-*1α 18SrRNA*-*3*
Ethylene	*EF*-*1α*	*EF*-*1α TUBα*
Cold	*18SrRNA*-*3*	*18SrRNA*-*3 UBI*
Heat	*EF*-*1α*	*EF*-*1α 18SrRNA*-*1 ADP*
Tissues	*TUBα*	*TUBα UBI TUBβ6*

#### Phytohormone treatments

Observation of comparative ΔCt, BestKeeper, Normfinder and geNorm indicated that the most stable candidate genes across ABA treatment were *UBI*, *18SrRNA*-*1* and *18SrRNA*-*3* while the least stable genes were *TUBβ6*, *GAPDH* and *ACT*. The optimal reference genes across GA_3_ treatment were *EF*-*1α*, *18SrRNA*-*3* and *UBI* according to comparative ΔCt, Normfinder and geNorm and the least stable reference genes were *TUBα*, *TUBβ6* and *ACT*. In the ethylene treatment, the preferred genes were *EF*-*1α*, *TUBα* and *UBI* according to comparative ΔCt, BestKeeper and Normfinder while *CYP2*, *18SrRNA*-*3* and *ACT* were the least stable (Fig. 4d–f; Additional file 2: Table S1).

According to RefFinder, the order of reference gene stability under ABA treatment was: *UBI* > *18SrRNA*-*1* > *18SrRNA*-*3* > *EF*-*1α* > *CYP2* > *ADP* > *HSP90* > *TUBα *> *TUBβ6* > *GAPDH *> *ACT*. The ranking of gene stability across GA_3_ treatment was: *EF*-*1α *> *18SrRNA*-*3 *>* UBI* > *HSP90* > *18SrRNA*-*1* > *CYP2* > *GAPDH *> *ADP* > *TUBα* > *TUBβ6* > *ACT*. Under ethylene treatment the ranking of reference gene stability was: *EF*-*1α *> *TUBα *> *UBI *> *TUBβ6* >* HSP90* > *GAPDH* > *18SrRNA*-*1* > *ADP* > *CYP2 *> *18SrRNA*-*3 *> *ACT* (Fig. 5d–f). According to value of Vn/n + 1 < 0.15 indicates that the supplemental reference genes will not manifestly change the normalization, the geNorm data predicted that the pair-wise value of V3/4 was < 0.15 in the ABA treatment. In the GA_3_ and ethylene treatments the pair-wise values of V2/3 were < 0.15 (Fig. 6). Therefore, *UBI*, *18SrRNA*-*1* and *18SrRNA*-*3* were considered stable candidate genes across the tested ABA treatments while *EF*-*1α* and *18SrRNA*-*3* were determined to be suitable reference genes under GA_3_ treatment and *EF*-*1α* and *TUBα* were found to be the most suitable reference genes under ethylene treatment (Table 2).

#### Temperature stimuli

Based on the gene stability data, which were calculated by comparative ΔCt, BestKeeper, Normfinder and geNorm, *18SrRNA*-*3*, *UBI* and *ADP* were found to be the most stable genes in cold treatment according to comparative ΔCt, Normfinder and geNorm while *TUBβ6*, *TUBα* and *ACT* were the least steady across cold treatment. Under heat stress, *EF*-*1α*, *18SrRNA*-*1* and *ADP* showed the best stability according to comparative ΔCt, Normfinder and geNorm while *HSP90*, *TUBα* and *ACT* showed the least stability (Fig. 4g, h; Additional file 2: Table S1).

According to RefFinder, the order of reference gene stability under cold treatment was: *18SrRNA*-*3* >* UBI* > *ADP* > *HSP90* > *GAPDH* > *CYP2* > *EF*-*1α* > *18SrRNA*-*1* > *TUBβ6 *> *TUBα* > *ACT*. Gene stability under heat stress was ranked: *EF*-*1α* >* 18SrRNA*-*1* > *ADP* > *GAPDH* > *18SrRNA*-*3* >* CYP2* >* TUBβ6* > *UBI* >* HSP90* > *TUBα* > *ACT* (Fig. 5g, h). The geNorm analysis data predicted that the pair-wise values of V2/3 were < 0.15 in cold treatment and that the pair-wise values of V3/4 were below the cut-off value of 0.15 under heat stress (Fig. 6). This suggests that *18SrRNA*-*3* and *UBI* was the best combination of reference genes under cold treatment and *EF*-*1α*, *18SrRNA*-*1* and *ADP* were the most suitable reference genes for *H. brevisubulatum* under heat stress (Table 2).

#### Shoot and root tissue

The comparative ΔCt, BestKeeper and Normfinder results indicated that *TUBα* and *UBI* were two of the three most stably expressed genes in shoot and root tissue, while geNorm results indicated that the two most stable genes were *TUBα* and *TUBβ6*. The least stable genes between shoot and root tissue were *GAPDH*, *HSP90* and *CYP2* (Fig. 4i; Additional file 2: Table S1).

Based on RefFinder, the ranking order of reference gene stability between shoot and root tissue was: *TUBα* > *UBI* > *TUBβ6* >* EF*-*1α* > *ACT *> *18SrRNA*-*3* > *ADP* > *18SrRNA*-*1 *> *GAPDH* >* HSP90* > *CYP2* (Fig. 5i). The geNorm data analysis showed that the pair-wise value of V3/4 was < 0.15 (Fig. 6). Therefore, *TUBα*, *UBI* and *TUBβ6* were considered the most appropriate reference genes between shoot and root tissue in *H. brevisubulatum* (Table 2). Notably, the study found that the expression of *TUBα* and *TUBβ6* in roots was unstable and decreased over time under heat stress while the expression of *TUBβ6* in roots increased over time under ABA treatment. However, the expression of *UBI* in eight treatments was stable in both shoots and roots. Therefore, *UBI* is more adaptable and stable than *TUBα* and *TUBβ6* following abiotic stress.

## Discussion

The qRT-PCR approach has become a key method for gene expression profiling owing to its accuracy, sensitivity and efficiency [37]. It is crucial to select reference genes that are stably expressed amongst treatment groups in qRT-PCR studies; a good reference gene should maintain invariable expression levels in different tissues, organs and developmental stages, as well as under various stress conditions [38]. Therefore, to select appropriate reference genes under specific conditions must be statistically and experimentally helpful for biological technicians.

RNA quantity, primer amplification efficiency and specificity are important for qRT-PCR analysis [39]. Here, the OD ratio (A_260_/A_280_) of all RNA samples was between 1.8 and 2.0 and the amplification efficiency of the 11 candidates ranged from 88 to 115% (all R^2^ > 0.990) (Table 1). Thus, the quality of the RNA and amplification was sufficient for qRT-PCR (Fig. 1b). Previous studies have reported that the expression level of reference genes is not always stable under all experimental conditions [27–30] and that mRNA expression levels varied among several housekeeping genes [40, 41]. Here these factors were confirmed in different tissues of *H. brevisubulatum* under various abiotic stresses and hormone treatments (Fig. 3).

Thus far, several reports have shown the importance of selecting proper reference genes for data normalization and have highlighted the identification of these genes vary depending on the model of study [6, 7, 26, 27, 42, 43]. Although reference genes have been identified in barley (*H. vulgare*) [26, 28, 29, 32], there are no reports about a systematic and comprehensive study on the selection and identification of reliable reference genes for *H. brevisubulatum* under various conditions. In the current study, we tested the expression stabilities of 11 candidate reference genes under eight stress and phytohormone conditions at different time points in shoot and root tissues (Additional file 2: Table S1). We have selected these 11 reference genes by referring to existing research reports: *ADP*, *UBI*, *ACT*, *GAPDH* and *HSP90* were reported in barley [32]; *18SrRNA*, *TUBα* and *TUBβ6* were reported in *Corchorus capsularis* [27]; and *EF*-*1α* and *CYP2* were reported in *Pennisetum glaucum* and *Lycoris aurea* [25, 44]. We also analyzed large-scale transcriptome data of *H. brevisubulatum* to help select these candidate reference genes. Using three different software programs (geNorm, Normfinder and BestKeeper) [17, 23, 24], the 11 candidate reference genes were found to exhibit differences in their stability under eight stress and phytohormone treatments in *H. brevisubulatum* (Fig. 3; Additional files 1 and 2: Fig. S1 and Table S1). Additionally, because of the different algorithms, the rankings generated by the three software programs and ∆Ct were not completely identical (Additional file 2: Table S1). For example, in the NaCl stress subset, *ADP* and *UBI* were ranked as the most stable by ∆Ct, Normfinder and BestKeeper while geNorm identified *EF*-*1α* and *18SrRNA*-*3* as the most stable. In the GA_3_ stress subset and cold stress subset, *EF*-*1α* and *18SrRNA*-3, which were the most stable genes identified by ∆Ct, Normfinder and geNorm, were ranked at a medium position in BestKeeper (Additional file 2: Table S1). This apparent divergence is probably because of discrepancies in the four statistical algorithms to calculate stability; similar situations have occurred in other studies [27, 44, 51].

The geNorm program was used to determine the stability of a candidate gene by pairwise variations and the Normfinder and BestKeeper programs were used to prevent co-regulation and to further assess the analysis results obtained from the geNorm program. According to the geNorm algorithm, reference genes with an M value below 1.5 were considered to be stably expressed and the optimal number of reference genes was determined based on the pairwise variation between sequential ranked genes (Vn/Vn + 1) with the cut-off value of 0.15. When the Vn/n + 1 value was below 0.15, no additional genes were required for accurate normalization [23]. According to this study, the V2/3 values were below 0.15 in NaCl, PEG6000, mannitol, GA_3_, ethylene and cold, suggesting that adding an extra gene for normalization was not necessary to obtain more accurate results. For ABA, heat and tissue treatments, the V2/3 values were 0.152, 0.165 and 0.166, indicating that additional reference genes may be required (Fig. 6). Previous reports showed that the variation of reference genes was relatively large [44–47]; here, the V3/4 value of the ABA, heat and tissue treatments was less than 0.15, indicating that the variation of these 11 reference genes was relatively small under various stress conditions in *H. brevisubulatum* (Fig. 6). Considered to be an integrative statistical program, RefFinder has been widely applied to evaluate the overall stability of reference gene expression and determine appropriate reference genes for diverse plant species [31, 48]. Based on the comprehensive RefFinder analysis, we have summarized and demonstrated the ordering of reference gene stability under various treatments and tissues (Fig. 5). Comprehensive analysis of the ordering performed by RefFinder and the numbers produced by geNorm was used to identify the most stable reference gene or combination of genes in each treatment and tissue (Table 2).

Wild barley is a wild germplasm resource with excellent resistance to abiotic stress such as salt tolerance [1, 50]. Therefore, it is necessary to strengthen the research on the expression and function of genes in *H. brevisubulatum*. This study is the first systematic and comprehensive description of suitable and stable reference genes for *H. brevisubulatum* in different tissues under various stress conditions and has provided a selection of reference genes that can be used to accurately examine gene expression in subsequent research in *H. brevisubulatum*. In summary, 11 reference gene candidates were selected based on our transcriptome sequence data and previous reports and their expression stability was assessed using four algorithms. The candidates were then ranked according to their stability to determine the most suitable reference gene and gene combination for each treatment (Table 2). Most of the previous reports have studied appropriate combinations of reference genes and the identification of single reference genes suitable for certain stress conditions is rarely reported [49]. In fact, it is often difficult to standardize qRT-PCR data when using a combination of reference genes. To ensure the accuracy of the experiment and simplify the experimental operation, we also proposed a standardization method for single reference genes (Table 2). Furthermore, *EF*-*1α* and *UBI* can be applied to any of the stress treatments and tissues (Table 2) as their expression did not differ significantly in shoot and root tissue under eight stress and phytohormone treatments (Fig. 3; Additional file 1: Fig. S1), revealing the importance and stability of these two reference genes under multiple stress conditions in *H. brevisubulatum*.

## Conclusions

In this study, our main contribution was to identify reference genes with suitable and stable expression in wild barley under various stress conditions and in different tissues to provide a useful resource for future studies. 11 candidate reference genes were screened from the wild barley transcriptome database, and their stability was evaluated and ranked based on four algorithms (Normfinder, BestKeeper, geNorm and the comparative ∆Ct method). We identified the most stable single reference genes for each treatment: ADP for NaCl treatment; EF-1α for PEG, GA_3_, ethylene and heat stress; UBI for mannitol and ABA stress; 18SrRNA-3 for cold stress; TUBα for different tissues. We have also proposed the most suitable reference gene combinations: ADP and UBI in NaCl stress; EF-1α and UBI in PEG and mannitol stress; UBI, 18SrRNA-1 and 18SrRNA-3 in ABA treatment; EF-1α and 18SrRNA-3 in GA_3_ treatment; EF-1α and TUBα in ethylene treatment; 18SrRNA-3 and UBI in cold stress; EF-1α, 18SrRNA-1 and ADP in heat stress; and TUBα, UBI and *TUBβ6* in different tissues. Our results demonstrate the importance of transcriptome data as a useful resource for the screening of candidate reference genes and highlight the need for specific reference genes for specific conditions. Furthermore, the reference genes selected in the current study will be helpful for accurate normalization of qRT-PCR data and will facilitate future gene expression and functional verification studies in *H. brevisubulatum*.

## Methods

### Plant material and treatments

Seeds of *H. brevisubulatum* were collected from the saline grassland in the suburbs of Hohhot of Inner Mongolia Autonomous Region of China and obtained this material through salt tolerance screening. A large number of individual plants have been obtained through tissue culture, and reserved plenty of seeds for subsequent research.

The wild barley seeds were immersed in water for 2 d at 4 °C for vernalization, then placed in an incubator at 22–25 °C (night and day thresholds) under a 16 h/8 h photoperiod for 2–3 d to promote seed germination. When the seedlings were about 1.0 cm long, they were transferred to a 250 ml beaker containing Hoagland’s nutrient solution. The seedling roots were immersed in the nutrient solution and the young shoots floated on gauze on the surface. The seedlings were then cultured for approximately 2 w until the plants grew to the two-leaf and one-heart stage at which point various abiotic stress treatments: 350 mM NaCl, 350 mM mannitol, 10% PEG6000, 20 μM ABA, 100 μM GA_3_, 100 μM ethylene, cold (4 °C) and heat stress (42 °C) were performed.

### Sample collection and RNA extraction

Shoot and root tissues of *H. brevisubulatum* for the various abiotic stress treatments (salt, mannitol, PEG6000, ABA, GA_3_, ethylene, cold and heat stresses) were collected and labelled at 0 h, 0.5 h, 1 h, 2 h, 3 h, 6 h and 12 h with four biological replicates taken for each. No 12 h samples were taken for the ethylene treatment. To ensure the integrity of the sample RNA, isolated samples were immediately frozen in liquid nitrogen and stored at − 80 °C before RNA extraction.

Total RNA was extracted using Trizol lysis (Takara, Dalian, China). First, frozen specimens were ground in liquid nitrogen to a fine powder with a pestle and a mortar. Next, the powder was completely dissolved in Trizol reagent blended by vortexing. Then, the mixture was centrifuged at 12,000×*g* at 4 °C for 5 min. Phenol–chloroform equal to one-fifth the total volume was added to the supernatant to purify the RNA and the samples were centrifuged at 12,000×*g* at 4 °C for 5 min. An equal volume of chloroform was added and aspirated to remove the phenol before a half volume of 8 M LiCl and a half volume of 75% alcohol was added to the supernatant to precipitate the RNA for at least 1 h. Finally, the RNA precipitate was washed twice with 75% ethanol and dissolved with RNA-free water before the RNA quality and concentration were measured. The integrity and purity of the RNA samples were determined by 1.5% agarose gels electrophoresis and the RNA concentration was assessed by a Thermo Scientific NanoDrop 2000c UV–Vis spectrophotometer (Thermo Fisher Scientific Inc., Waltham, MA, USA).

### cDNA synthesis

cDNAs were reverse transcribed from total RNA using a HiScript II Q Select RT SuperMix reagent Kit (Vazyme Biotech Co., Ltd, Nanjing, China) following the manufacturer’s protocol. This reverse transcription kit removes trace amounts of DNA from total RNA to ensure that qRT-PCR amplification is completely derived from cDNA. The reverse-transcribed cDNA templates were diluted 1:5 with nuclease-free water and stored at − 20 °C until qRT-PCR analysis with minimal thawing and refreezing.

### Specific primer design

We examined the reference genes of barley (*H. vulgare*) in the internal control genes (ICG) database that is a wiki-based knowledgebase of internal control genes (or reference genes) for RT-qPCR normalization in a variety of species (http://icg.big.ac.cn/index.php/Main_Page) such as ADP, UBI, ACT, GAPDH and HSP90. Moreover, we also referred to previous research in *L. aurea* (EF-1α, CYP2) [44] and *Corchorus capsularis* (18SrRNA, TUBα, TUBβ6) [27]. Then, the NCBI local blast software (blast-2.7.1+) was used to compare the sequences of reference genes of different species with existing wild barley transcriptome databases and the sequences of 11 candidate reference genes of wild barley were obtained. The protein prediction was performed using the open reading frame (ORF) finder (http://www.ncbi.nlm.nih.gov/gorf/). Multiple alignments of predicted amino acid sequences were created using DNAMAN [33].

Specific primers were designed using the Primer-BLAST tool in NCBI (available online: (http://www.ncbi.nlm.nih.gov/tools/primer-blast/) based on the sequences of the candidate reference genes. The parameters were as follows: product size was 70–200 bp; primer melting temperature (T_m_) was 58–62 °C; database was nr; organism was *H. brevisubulatum* (taxid: 52155). To obtain the most suitable primers, primer 5 was used to check the primer mismatch, hairpin structure and dimer energy value before 2–3 pairs of primers were designed per gene and synthesized by Sangon Biotech Co., Ltd. (Shanghai, China).

### Primer-specific detection and amplification efficiency

It was necessary to detect the specificity of the primer by gel electrophoresis and melting curve analysis. The efficiency reflects the adequacy of the qRT-PCR reaction, the pros and cons of the primers, and the quality of the template [34]. The efficiencies (E) and correlation coefficients (R^2^) were calculated for each reference gene [35]. The standard curve of each primer pair was established with serial dilutions of cDNA ((1/5)^0^, (1/5)^1^, (1/5)^2^, (1/5)^3^ and (1/5)^4^). The amplification efficiency (E) of qRT-PCR was determined according to the equation: E = 10 ^1/K^, where K represents the slope of the standard curve.

### Quantitative real-time PCR (qRT-PCR)

qRT-PCR was performed using TB Green™ Premix Ex Taq™ II (Takara, Dalian, China) on the StepOne Plus Real-Time PCR System (Applied Biosystems, Foster City, CA, USA). Twenty microliter-reactions were performed in MicroAmp Fast Optical 96-Well reaction plates with barcodes (Applied Biosystems, Foster City, CA, USA) with each reaction containing 2 μl cDNA, 10 μl 2 × qPCR Mix, 0.4 μl 50 × ROX Reference Dye, 0.8 μl each of the forward and reverse primers, and 6 μl of nuclease-free water. The PCR program involved a two-step process of initial denaturation at 95 °C for 30 s followed by 40 cycles of denaturation at 95 °C for 5 s and annealing at 60 °C for 30 s where the fluorescence signal was detected. Four technical replicates were included for each reaction. Melting curve data were gathered from 95 °C maintain 15 s down to 60 °C hold 1 min, then up to 95 °C keep 15 s (with increments of 0.3 °C), with fluorescence signals detected during this increase.

### Statistical analysis

Excel-based programs were used to analyze gene expression stability, including geNorm, NormFinder and BestKeeper. RefFinder, which is a user-friendly web-based comprehensive tool developed for evaluating and screening reference genes from extensive experimental datasets (http://150.216.56.64/referencegene.php), was also used. It integrates the currently available major computational programs (geNorm, Normfinder, BestKeeper and the comparative ∆C_t_ method) to compare and rank the tested candidate reference genes. Based on the rankings from each program, it assigned an appropriate weight to an individual gene and calculated the geometric mean of their weights for the overall final ranking [31]. Finally, the most suitable internal reference gene under each treatment condition was determined. All assays were repeated at least three times and the data represent the mean ± SD. Microsoft^®^ Excel 2016 and SAS 9.2 statistical software were used for data analysis and Duncan’s Multiple Range Test, respectively.

## Additional files

**Additional file 1: Fig. S1.** Variation in the expression of reference genes using distribution of cycle threshold (Ct) values in line charts.

**Additional file 2: Table S1.** Expression stability of the 11 candidate reference genes under various stress treatments in *H. brevisubulatum.*
