# Supplementary figures and images for: Reference genes identification for normalization of qPCR under multiple stresses in Hordeum brevisubulatum
Source: Plant Methods. 2018 Dec 18;14:110. doi: 10.1186/s13007-018-0379-3 (PMC6297944; doi:10.1186/s13007-018-0379-3)

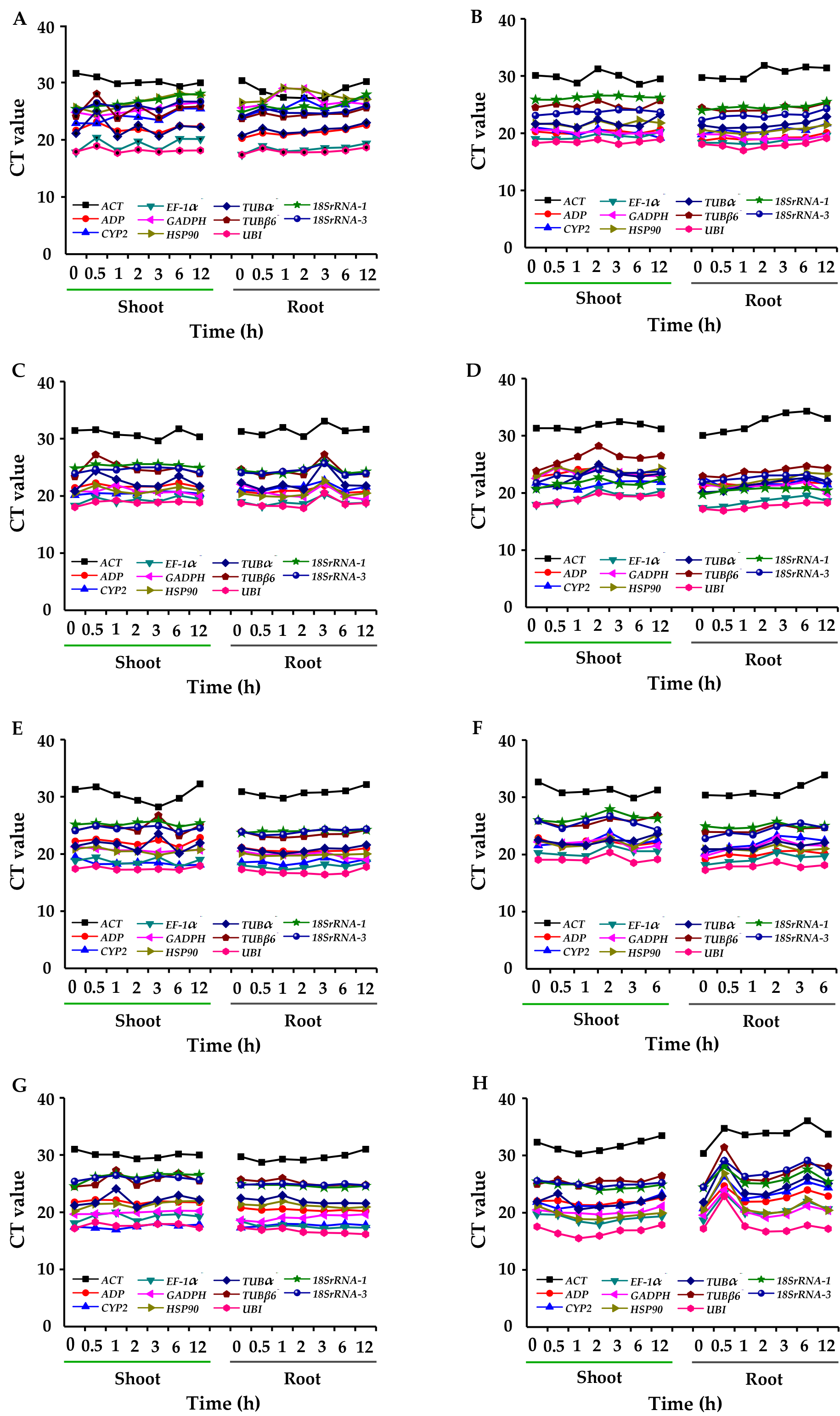

Supplement: Supplementary file 1 — Additional file 1: Fig. S1. Variation in the expression of reference genes using distribution of cycle threshold (Ct) values in line charts. [file 13007_2018_379_MOESM1_ESM.tif]
