# Supplementary material for: Reference genes identification for normalization of qPCR under multiple stresses in Hordeum brevisubulatum
Source: Plant Methods. 2018 Dec 18;14:110. doi: 10.1186/s13007-018-0379-3 (PMC6297944; doi:10.1186/s13007-018-0379-3)
Supplement: Supplementary file 2 — Additional file 2: Table S1. Expression stability of the 11 candidate reference genes under various stress treatments in H. brevisubulatum. [file 13007_2018_379_MOESM2_ESM.docx]

**Table S1.** Expression stability of the 11 candidate reference genes under various stress treatments in *H. brevisubulatum*.

| **Treatments** | **Rank** | **∆Ct** | | **BestKeeper** | | **Normfinder** | | **GeNorm** | |
| --- | --- | --- | --- | --- | --- | --- | --- | --- | --- |
|  |  | **Gene symbol** | **Standard Value** | **Gene symbol** | **Stability Value** | **Gene symbol** | **Stability Value** | **Gene symbol** | **Stability Value** |
| NaCl | 1 | *ADP* | 1.000 | *UBI* | 0.310 | *UBI* | 0.254 | *EF-1α\|18SrRNA-3* | 0.424 |
|  | 2 | *UBI* | 1.033 | *ADP* | 0.589 | *ADP* | 0.354 |  |  |
|  | 3 | *EF-1α* | 1.036 | *18SrRNA-3* | 0.710 | *18SrRNA-3* | 0.489 | *ADP* | 0.459 |
|  | 4 | *18SrRNA-3* | 1.046 | *EF-1α* | 0.818 | *EF-1α* | 0.503 | *TUBβ6* | 0.565 |
|  | 5 | *TUBβ6* | 1.206 | *TUBα* | 0.851 | *18SrRNA-1* | 0.709 | *TUBα* | 0.602 |
|  | 6 | *18SrRNA-1* | 1.211 | *18SrRNA-1* | 0.861 | *TUBβ6* | 0.883 | *UBI* | 0.640 |
|  | 7 | *TUBα* | 1.230 | *TUBβ6* | 0.942 | *TUBα* | 0.892 | *18SrRNA-1* | 0.735 |
|  | 8 | *HSP90* | 1.595 | *HSP90* | 0.946 | *HSP90* | 1.342 | *HSP90* | 0.971 |
|  | 9 | *CYP2* | 1.653 | *GAPDH* | 0.982 | *CYP2* | 1.394 | *CYP2* | 1.114 |
|  | 10 | *GAPDH* | 1.749 | *ACT* | 1.097 | *GAPDH* | 1.560 | *GAPDH* | 1.222 |
|  | 11 | *ACT* | 1.764 | *CYP2* | 1.183 | *ACT* | 1.574 | *ACT* | 1.320 |
| PEG6000 | 1 | *EF-1α* | 0.559 | *UBI* | 0.425 | *UBI* | 0.217 | *ADP\| EF-1α* | 0.266 |
|  | 2 | *ADP* | 0.579 | *18SrRNA-3* | 0.455 | *EF-1α* | 0.221 |  |  |
|  | 3 | *UBI* | 0.579 | *GAPDH* | 0.465 | *ADP* | 0.281 | *UBI* | 0.353 |
|  | 4 | *TUBβ6* | 0.624 | *EF-1α* | 0.494 | *TUBβ6* | 0.322 | *TUBβ6* | 0.390 |
|  | 5 | *HSP90* | 0.654 | *TUBβ6* | 0.494 | *18SrRNA-3* | 0.407 | *TUBα* | 0.419 |
|  | 6 | *18SrRNA-3* | 0.669 | *CYP2* | 0.521 | *HSP90* | 0.423 | *HSP90* | 0.449 |
|  | 7 | *TUBα* | 0.696 | *TUBα* | 0.555 | *TUBα* | 0.458 | *18SrRNA-3* | 0.479 |
|  | 8 | *GAPDH* | 0.724 | *ADP* | 0.586 | *GAPDH* | 0.516 | *GAPDH* | 0.512 |
|  | 9 | *18SrRNA-1* | 0.808 | *HSP90* | 0.646 | *18SrRNA-1* | 0.687 | *18SrRNA-1* | 0.545 |
|  | 10 | *CYP2* | 0.924 | *18SrRNA-1* | 0.837 | *CYP2* | 0.760 | *CYP2* | 0.621 |
|  | 11 | *ACT* | 1.225 | *ACT* | 0.859 | *ACT* | 1.149 | *ACT* | 0.731 |

**Table S1.** *Cont*.

| **Treatments** | **Rank** | **∆Ct** | | **BestKeeper** | | **Normfinder** | | **GeNorm** | |
| --- | --- | --- | --- | --- | --- | --- | --- | --- | --- |
|  |  | **Gene symbol** | **Standard Value** | **Gene symbol** | **Stability Value** | **Gene symbol** | **Stability Value** | **Gene symbol** | **Stability Value** |
| Mannitol | 1 | *UBI* | 0.644 | *UBI* | 0.412 | *UBI* | 0.243 | *ADP\|18SrRNA-1* | 0.314 |
|  | 2 | *EF-1α* | 0.645 | *18SrRNA-3* | 0.457 | *HSP90* | 0.287 |  |  |
|  | 3 | *HSP90* | 0.653 | *EF-1α* | 0.495 | *EF-1α* | 0.292 | *18SrRNA-3* | 0.380 |
|  | 4 | *18SrRNA-3* | 0.681 | *ADP* | 0.511 | *18SrRNA-3* | 0.388 | *EF-1α* | 0.410 |
|  | 5 | *18SrRNA-1* | 0.716 | *CYP2* | 0.534 | *ADP* | 0.482 | *HSP90* | 0.437 |
|  | 6 | *ADP* | 0.719 | *18SrRNA-1* | 0.584 | *18SrRNA-1* | 0.487 | *UBI* | 0.455 |
|  | 7 | *TUBβ6* | 0.910 | *HSP90* | 0.605 | *TUBβ6* | 0.728 | *GAPDH* | 0.555 |
|  | 8 | *GAPDH* | 0.919 | *GAPDH* | 0.626 | *GAPDH* | 0.729 | *CYP2* | 0.640 |
|  | 9 | *ACT* | 0.948 | *ACT* | 0.680 | *ACT* | 0.763 | *ACT* | 0.701 |
|  | 10 | *CYP2* | 0.955 | *TUBβ6* | 0.915 | *CYP2* | 0.790 | *TUBβ6* | 0.752 |
|  | 11 | *TUBα* | 1.025 | *TUBα* | 1.059 | *TUBα* | 0.888 | *TUBα* | 0.802 |
| ABA | 1 | *UBI* | 0.747 | *CYP2* | 0.495 | *UBI* | 0.152 | *UBI\|18SrRNA-1* | 0.454 |
|  | 2 | *18SrRNA-1* | 0.786 | *18SrRNA-3* | 0.574 | *18SrRNA-1* | 0.315 |  |  |
|  | 3 | *EF-1α* | 0.810 | *18SrRNA-1* | 0.675 | *EF-1α* | 0.354 | *EF-1α* | 0.490 |
|  | 4 | *18SrRNA-3* | 0.838 | *UBI* | 0.771 | *18SrRNA-3* | 0.388 | *18SrRNA-3* | 0.499 |
|  | 5 | *ADP* | 0.898 | *EF-1α* | 0.797 | *HSP90* | 0.545 | *ADP* | 0.581 |
|  | 6 | *HSP90* | 0.908 | *HSP90* | 0.902 | *ADP* | 0.574 | *HSP90* | 0.628 |
|  | 7 | *TUBα* | 0.944 | *ADP* | 0.917 | *TUBα* | 0.649 | *TUBα* | 0.667 |
|  | 8 | *TUBβ6* | 1.067 | *ACT* | 1.003 | *TUBβ6* | 0.867 | *TUBβ6* | 0.712 |
|  | 9 | *GAPDH* | 1.163 | *TUBα* | 1.063 | *GAPDH* | 0.974 | *GAPDH* | 0.779 |
|  | 10 | *CYP2* | 1.282 | *GAPDH* | 1.125 | *CYP2* | 1.088 | *CYP2* | 0.880 |
|  | 11 | *ACT* | 1.523 | *TUBβ6* | 1.316 | *ACT* | 1.399 | *ACT* | 0.997 |

**Table S1.** *Cont*.

| **Treatments** | **Rank** | **∆Ct** | | **BestKeeper** | | **Normfinder** | | **GeNorm** | |
| --- | --- | --- | --- | --- | --- | --- | --- | --- | --- |
|  |  | **Gene symbol** | **Standard Value** | **Gene symbol** | **Stability Value** | **Gene symbol** | **Stability Value** | **Gene symbol** | **Stability Value** |
| GA_3_ | 1 | *EF-1α* | 0.594 | *CYP2* | 0.354 | *EF-1α* | 0.169 | *EF-1α\|18SrRNA-3* | 0.380 |
|  | 2 | *18SrRNA-3* | 0.641 | *UBI* | 0.399 | *UBI* | 0.280 |  |  |
|  | 3 | *UBI* | 0.646 | *18SrRNA-3* | 0.408 | *18SrRNA-3* | 0.289 | *UBI* | 0.446 |
|  | 4 | *HSP90* | 0.667 | *HSP90* | 0.425 | *HSP90* | 0.355 | *HSP90* | 0.462 |
|  | 5 | *18SrRNA-1* | 0.678 | *GAPDH* | 0.481 | *18SrRNA-1* | 0.420 | *18SrRNA-1* | 0.477 |
|  | 6 | *ADP* | 0.698 | *EF-1α* | 0.551 | *ADP* | 0.445 | *ADP* | 0.496 |
|  | 7 | *GAPDH* | 0.780 | *18SrRNA-1* | 0.632 | *GAPDH* | 0.566 | *GAPDH* | 0.528 |
|  | 8 | *TUBα* | 0.805 | *TUBα* | 0.731 | *TUBα* | 0.634 | *TUBα* | 0.585 |
|  | 9 | *TUBβ6* | 0.852 | *ADP* | 0.773 | *CYP2* | 0.696 | *TUBβ6* | 0.614 |
|  | 10 | *CYP2* | 0.888 | *TUBβ6* | 0.783 | *TUBβ6* | 0.723 | *CYP2* | 0.664 |
|  | 11 | *ACT* | 1.268 | *ACT* | 0.859 | *ACT* | 1.184 | *ACT* | 0.774 |
| Ethylene | 1 | *EF-1α* | 0.668 | *TUBα* | 0.647 | *EF-1α* | 0.269 | *TUBα\| TUBβ6* | 0.312 |
|  | 2 | *UBI* | 0.684 | *UBI* | 0.651 | *UBI* | 0.308 |  |  |
|  | 3 | *TUBβ6* | 0.709 | *GAPDH* | 0.676 | *TUBβ6* | 0.408 | *HSP90* | 0.398 |
|  | 4 | *TUBα* | 0.760 | *EF-1α* | 0.720 | *TUBα* | 0.466 | *EF-1α* | 0.470 |
|  | 5 | *HSP90* | 0.797 | *HSP90* | 0.723 | *HSP90* | 0.549 | *UBI* | 0.502 |
|  | 6 | *18SrRNA-1* | 0.801 | *CYP2* | 0.742 | *GAPDH* | 0.552 | *18SrRNA-1* | 0.533 |
|  | 7 | *GAPDH* | 0.825 | *TUBβ6* | 0.775 | *18SrRNA-1* | 0.565 | *ADP* | 0.598 |
|  | 8 | *ADP* | 0.895 | *18SrRNA-1* | 0.787 | *18SrRNA-3* | 0.667 | *GAPDH* | 0.652 |
|  | 9 | *18SrRNA-3* | 0.900 | *ACT* | 0.873 | *ADP* | 0.678 | *18SrRNA-3* | 0.695 |
|  | 10 | *CYP2* | 0.910 | *18SrRNA-3* | 0.905 | *CYP2* | 0.687 | *CYP2* | 0.729 |
|  | 11 | *ACT* | 1.382 | *ADP* | 1.018 | *ACT* | 1.279 | *ACT* | 0.848 |

**Table S1.** *Cont*.

| **Treatments** | **Rank** | **∆Ct** | | **BestKeeper** | | **Normfinder** | | **GeNorm** | |
| --- | --- | --- | --- | --- | --- | --- | --- | --- | --- |
|  |  | **Gene symbol** | **Standard Value** | **Gene symbol** | **Stability Value** | **Gene symbol** | **Stability Value** | **Gene symbol** | **Stability Value** |
| Cold | 1 | *18SrRNA-3* | 0.591 | *CYP2* | 0.245 | *18SrRNA-3* | 0.214 | *ADP\|18SrRNA-3* | 0.289 |
|  | 2 | *UBI* | 0.662 | *HSP90* | 0.419 | *UBI* | 0.382 |  |  |
|  | 3 | *ADP* | 0.664 | *GAPDH* | 0.467 | *ADP* | 0.418 | *UBI* | 0.342 |
|  | 4 | *HSP90* | 0.721 | *ACT* | 0.510 | *HSP90* | 0.459 | *EF-1α* | 0.408 |
|  | 5 | *GAPDH* | 0.727 | *UBI* | 0.518 | *GAPDH* | 0.480 | *18SrRNA-1* | 0.431 |
|  | 6 | *18SrRNA-1* | 0.737 | *18SrRNA-3* | 0.546 | *18SrRNA-1* | 0.544 | *GAPDH* | 0.504 |
|  | 7 | *EF-1α* | 0.741 | *TUBα* | 0.621 | *EF-1α* | 0.560 | *HSP90* | 0.573 |
|  | 8 | *TUBβ6* | 0.797 | *TUBβ6* | 0.674 | *TUBβ6* | 0.613 | *TUBβ6* | 0.630 |
|  | 9 | *TUBα* | 0.860 | *ADP* | 0.713 | *TUBα* | 0.707 | *TUBα* | 0.671 |
|  | 10 | *CYP2* | 0.879 | *EF-1α* | 0.864 | *CYP2* | 0.714 | *CYP2* | 0.715 |
|  | 11 | *ACT* | 0.947 | *18SrRNA-1* | 0.893 | *ACT* | 0.802 | *ACT* | 0.757 |
| Heat | 1 | *EF-1α* | 0.786 | *GAPDH* | 0.743 | *EF-1α* | 0.140 | *EF-1α\|18SrRNA-1* | 0.463 |
|  | 2 | *18SrRNA-1* | 0.906 | *ADP* | 0.760 | *18SrRNA-1* | 0.544 |  |  |
|  | 3 | *ADP* | 0.926 | *18SrRNA-1* | 0.827 | *ADP* | 0.586 | *ADP* | 0.518 |
|  | 4 | *18SrRNA-3* | 0.936 | *UBI* | 0.980 | *18SrRNA-3* | 0.603 | *18SrRNA-3* | 0.584 |
|  | 5 | *CYP2* | 0.960 | *EF-1α* | 1.007 | *CYP2* | 0.619 | *CYP2* | 0.671 |
|  | 6 | *TUBβ6* | 0.980 | *HSP90* | 1.228 | *TUBβ6* | 0.627 | *TUBβ6* | 0.745 |
|  | 7 | *GAPDH* | 1.083 | *18SrRNA-3* | 1.293 | *GAPDH* | 0.836 | *GAPDH* | 0.810 |
|  | 8 | *UBI* | 1.154 | *TUBβ6* | 1.360 | *UBI* | 0.933 | *UBI* | 0.889 |
|  | 9 | *HSP90* | 1.178 | *CYP2* | 1.430 | *HSP90* | 0.960 | *HSP90* | 0.938 |
|  | 10 | *TUBα* | 1.197 | *ACT* | 1.454 | *TUBα* | 0.974 | *TUBα* | 0.986 |
|  | 11 | *ACT* | 1.235 | *TUBα* | 1.679 | *ACT* | 1.054 | *ACT* | 1.031 |

**Table S1.** *Cont*.

| **Treatments** | **Rank** | **∆Ct** | | **BestKeeper** | | **Normfinder** | | **GeNorm** | |
| --- | --- | --- | --- | --- | --- | --- | --- | --- | --- |
|  |  | **Gene symbol** | **Standard Value** | **Gene symbol** | **Stability Value** | **Gene symbol** | **Stability Value** | **Gene symbol** | **Stability Value** |
| Tissues | 1 | *TUBα* | 1.259 | *UBI* | 0.483 | *UBI* | 0.562 | *TUBα\| TUBβ6* | 0.266 |
|  | 2 | *UBI* | 1.265 | *TUBα* | 0.557 | *ACT* | 0.576 |  |  |
|  | 3 | *EF-1α* | 1.275 | *TUBβ6* | 0.595 | *TUBα* | 0.735 | *EF-1α* | 0.431 |
|  | 4 | *ACT* | 1.275 | *EF-1α* | 0.608 | *EF-1α* | 0.750 | *UBI* | 0.560 |
|  | 5 | *TUBβ6* | 1.293 | *ACT* | 0.716 | *TUBβ6* | 0.796 | *ACT* | 0.690 |
|  | 6 | *18SrRNA-3* | 1.502 | *ADP* | 0.896 | *18SrRNA-3* | 1.049 | *18SrRNA-3* | 0.828 |
|  | 7 | *ADP* | 1.538 | *18SrRNA-3* | 0.982 | *ADP* | 1.067 | *ADP* | 0.928 |
|  | 8 | *18SrRNA-1* | 1.891 | *18SrRNA-1* | 1.122 | *18SrRNA-1* | 1.602 | *18SrRNA-1* | 1.089 |
|  | 9 | *GAPDH* | 1.921 | *HSP90* | 1.319 | *GAPDH* | 1.620 | *GAPDH* | 1.321 |
|  | 10 | *HSP90* | 1.973 | *GAPDH* | 1.387 | *HSP90* | 1.675 | *HSP90* | 1.464 |
|  | 11 | *CYP2* | 2.017 | *CYP2* | 1.440 | *CYP2* | 1.731 | *CYP2* | 1.564 |

Note: Treatments were eight abiotic stresses and a different tissues; rank is decreasing gene stability based on four algorithms; ∆Ct is the average of the standard deviation of reference genes between different time points; BestKeeper is an algorithm to determine the correlation between samples and the 11 reference genes; Normfinder is an algorithm for identifying the optimal normalization gene among the 11 candidate reference genes according to their expression stability; geNorm is a popular algorithm to determine the most stable reference (housekeeping) genes and the number of reference genes required for standardized samples.
